# Retraction: The absence of MyD88 has no effect on the induction of alternatively activated macrophage during *Fasciola hepatica *infection

**DOI:** 10.1186/1471-2172-13-3

**Published:** 2012-01-16

**Authors:** HongLin Luo, Weiyi Huang, Dongying Wang, Haoju Wang, Kui Nie

**Affiliations:** 1 Laboratory of Infection & Immunology Research, College of Animal Science & Technology, Southwest University, Chongqing, China; 2 Parasitology department, College of Animal Science & Technology, Guangxi University, Nanning, China; 3 ENVA, UMR BIPAR, Ecopham, Ecole Nationale Vétérinaire d'Alfort, Maisons-Alfort, France

## Retraction

The authors would like to retract the article "The absence of MyD88 has no effect on the induction of alternatively activated macrophage during *Fasciola hepatica *infection" published in BMC Immunology (2011, **12**:63). The text and figures in this article [1] have been misappropriated from a different set of experiments using a different parasite, conducted in the laboratory of Prof J Allen and presented in the thesis of K Mylonas. Lead author Dr HongLin Luo accepts full responsibility for this and would like to apologise to colleagues in the lab, the co-authors, Editors and readers. The other authors take no responsibility for the misappropriation.
